# Predictors of Opiate Utilization in the Treatment of Headache and Impact on Three-Month Outcomes Following Subarachnoid Hemorrhage

**DOI:** 10.7759/cureus.20773

**Published:** 2021-12-28

**Authors:** Dana Klavansky, Sheshali Wanchoo, Amanda Lin, Richard E Temes, Tania Rebeiz

**Affiliations:** 1 Department of Neurosurgery, North Shore University Hospital, Northwell Health, Manhasset, USA; 2 Department of Pharmacy and Neurocritical Care, North Shore University Hospital, Northwell Health, Manhasset, USA

**Keywords:** subarachnoid hemorrhage, chronic and acute pain management, outpatient care, average length of hospital stay, neurology and critical care, opiate use, refractory headache

## Abstract

Despite multiple investigational drugs, headache due to subarachnoid hemorrhage (SAH) remains inadequately controlled and requires high opiate utilization. This study investigates the factors associated with increased opiate usage for the management of headache in SAH in the first 14 days of admission, the association between opiate usage and hospital length of stay, and the incidence of opiate consumption during the outpatient follow up.

This is a single-center cross-sectional study. A total of 138 patients admitted between January 1, 2017, and May 31, 2019, with a diagnosis of SAH, were identified through a neurocritical care dashboard. Outpatient electronic medical records were evaluated at three months. Statistical analysis included descriptive statistics, Mann-Whitney U test, stepwise regression, and multiple regression analysis.

We found that of 138 patients, the majority (90%) were prescribed opiates during their hospitalization, and the mean daily morphine equivalent dosage was 18.74 mg. Steroid usage was associated with an increase in 14-day opiate usage (r = 0.4, p = 0.0001); however, the cerebral spinal fluid profile did not show a statistically significant correlation. Over 14 days, smokers significantly used more opiates compared to nonsmokers (353 mg vs. 184 mg, p = 0.01). In addition, peri-mesencephalic SAH required less morphine compared to aneurysmal SAH (195 mg vs. 283 mg, p = 0.004). Aneurysm clipping was associated with less opiate usage compared to aneurysm coiling (186 vs. 320, p = 0.08).

Only the high Hunt and Hess scale score predicted opiate usage, and the high modified Fisher scale score, aneurysmal SAH, and more opiate usage predicted hospital length of stay. A total of 48 patients (42%) suffered from headaches during their outpatient follow-up within three months of discharge; however, only six (5%) were still on opiates. There was a significant association between the amount of opiate used in the first 14 days of admission and the rate of post-discharge headache.

In summary, even though patients admitted with SAH require a large amount of opiate for headache management, this did not lead to more opiate consumption in the outpatient setting. However, patients continued to suffer from headaches at three months follow-up. This high opiate consumption is associated with increased hospital length of stay. Studies are needed to identify opiate sparing analgesics that target the pathogenesis of headaches in this patient population.

## Introduction

Subarachnoid hemorrhage (SAH) is a leading cause of mortality and morbidity, often presenting with “the worst headache of life.” The headache can persist for days to weeks and is a cause of significant disability [1,2]. In their prospective study, Morad et al. [3] showed that continuous usage of opiates and acetaminophen failed to adequately attenuate severe headache in SAH patients, with no significant decline in usage during the hospital length of stay [3]. In addition, inadequately controlled headache is one of the frequent causes of readmission, leading to an increase in healthcare costs [4]. Moreover, a recent case-control study revealed an association between opiate addiction and aneurysmal SAH [5].

While headache due to SAH is a major cause of disability, there are few studies and no guidelines on its management. This could be due to a lack of understanding of its pathogenesis, natural history, contributing factors, and limited therapeutic interventions [6,7]. Headache due to SAH is thought to be due to an inflammatory cascade caused by meningeal irritation from blood products and upregulation of N-methyl-D-aspartate (NMDA) receptors [8]. Antagonists of NMDA receptors, such as steroids and magnesium, have been investigated as therapeutic options. However, despite the use of high-dose dexamethasone, pain often remains inadequately controlled and opiate usage remains very high [6]. We sought to evaluate predictors of opiate usage in headache following SAH and the association between inpatient opiate utilization and outpatient opiate dependence.

The objectives of our study are to investigate the factors associated with increased opiate usage in the first 14 days of admission, to evaluate the association between opiate usage in the first 14 days and hospital length of stay, and to measure the incidence of opiate usage during the outpatient follow up.

## Materials and methods

Patient selection

This is a single-center cross-sectional study approved by the Northwell Institutional Review Board. Consecutive patients admitted between January 1, 2017, and May 31, 2019, with a diagnosis of SAH, were identified retrospectively through a neurocritical care dashboard. Patients’ charts were reviewed by two independent reviewers (authors DK and SW). Patients older than 18 years of age with Hunt and Hess (HH) grades 1-3 or higher HH grades (4 or 5) whose mentation improved within 48 hours after admission were included in the study. Exclusion criteria included opiate use prior to SAH, pregnancy, and the inability to verbalize pain needs, including prolonged intubation greater than 48 hours.

Data collection

Baseline demographic data such as age, gender, race and ethnicity, and smoking status were abstracted through chart review. Admission predictors of headache severity such as the Hunt and Hess scale (HHS), modified Fisher scale (mFS), and neuroimaging data were analyzed. Cerebrospinal fluid (CSF) white blood cells, red blood cells, protein, and lactate levels were collected. Exposure to analgesics, such as steroids, was tabulated and the total morphine equivalent dosage used during the entire hospitalization was calculated. The outpatient and inpatient electronic medical records were evaluated at three months follow-up or at the time of readmission for the presence of headache and the continued outpatient utilization of opiates.

Statistical analysis

Descriptive statistics for continuous variables and percentages were calculated for categorical variables. Mann-Whitney U test was used to compare differences between the variables. Pearson correlation coefficient was used to evaluate the correlation between predictors of opiate utilization during the first 14 days of admission and length of stay in the hospital, and the rate of post-discharge headache. Stepwise regression analysis was used to assess factors that predict opiate usage in the first 14 days of admission. In addition, multiple regression analysis was utilized to test the hypothesis that daily morphine equivalent dosage (DMED) is a predictor of increased hospital length of stay. Stepwise logistic regression was used to test the hypothesis that DMED was significantly associated with predicting opiate usage and predictors associated with hospital length of stay.

## Results

**Figure 1:**
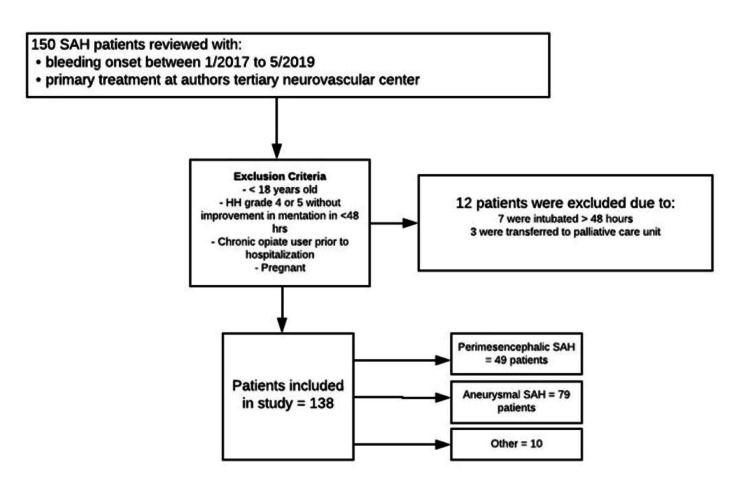
Flow diagram for SAH patients. SAH: subarachnoid hemorrhage; HH: Hunt and Hess.

During the study period, we retrospectively identified 150 patients with SAH. Twelve patients were excluded. A total of 138 cases with SAH were analyzed, of which 79 were aneurysmal and 49 were peri-mesencephalic (Figure 1).

The mean age of the patients was 52.6 years (range = 20-95 years). There was an almost equal distribution between genders (68 men and 70 women). Most of the patients had high mFS, with mFS 3 and 4 constituting 66% of the patient population. There was an almost equal number of clipped vs. coiled aneurysms (29.1% vs. 27%, respectively). On average, the length of ICU stay was 10 days, and the length of hospital stay was 16 days (Table 1).

**Table 1:** Patient information. mFS: modified Fischer scale.

Patient information	
Demographic (no, %)	American Indian or Alaska Native (1, 0.7%)
	Asian (17, 11/4%)
	Black or AA (39, 26.2%)
	Native Hawaiian or Pacific Islander (3, 2%)
	White (46, 30/9%)
	Hispanic or Latino (22, 14.8%)
	Multiracial (36, 24.2%)
	Other (7, 4.7%)
Average age (year, range)	52.66 (20-95)
Male sex (no, %)	68 (45.3%)
Smoking – yes/no (no, %)	Yes, current (15, 10.2%)
	No (72, 49%)
	Unknown (56, 38.1%)
	Previous smoker (4, 2.7%)
Clipping vs. coiling (no, %)	Clipped (43, 29.1%)
	Coiled (40, 27%)
	Webb’ed (2, 1.4%)
	Other (5, 3.4%)
mFS score (no, %)	1 (43, 29.1%)
	2 (7, 4.7%)
	3 (55, 37.2%)
	4 (43, 29.1%)
Length of ICU stay, mean days (range)	10 (2-30)
Length of hospital stay, mean days (range)	16 (2-50)
Disposition – yes/no, outpatient follow up (no, %)	Yes, outpatient follow up (124, 83.2%)
	No, outpatient follow up (25, 16.8%)

The majority of patients (121, 90%) were prescribed opiates during their hospitalization. Oxycodone was the most commonly prescribed opiate (oral morphine equivalent [OME] total: 22,951 mg), followed by tramadol (total OME: 6371 mg), Dilaudid (total OME: 6210.5 mg), fentanyl (total OME: 3396.3 mg), and morphine (total OME: 546 mg).

**Figure 2:**
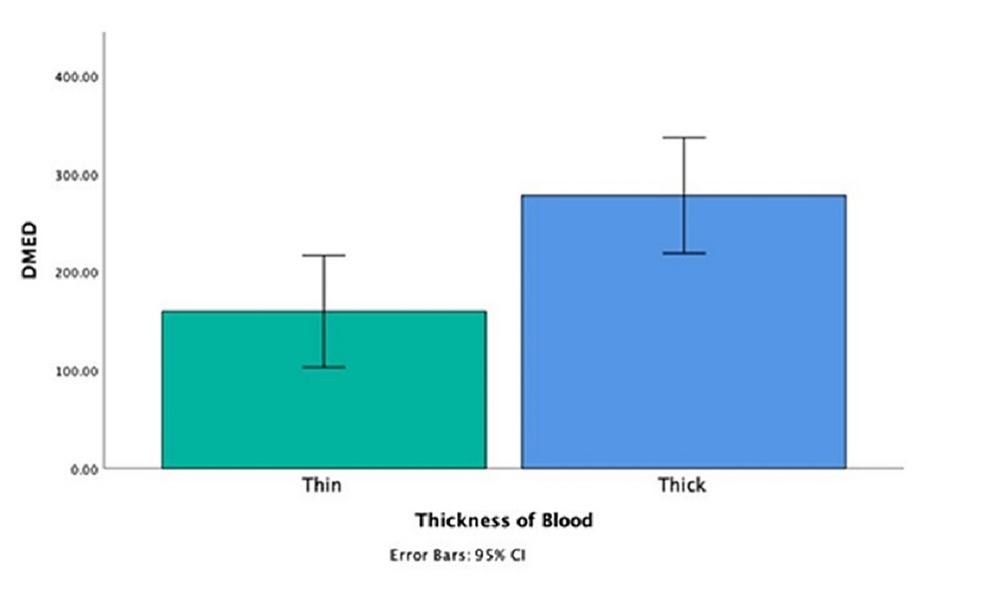
Daily morphine equivalence vs. thickness of blood. DMED: daily morphine equivalence dosage.

The median DMED per patient utilized while hospitalized was 142.5 mg (min = 0, max = 1264.75 mg). There was no significant difference in the 14 days opiate usage in terms of gender, race, presence of hydrocephalus, or intraventricular hemorrhage (IVH) on imaging. There was a weak negative correlation between opiate use over 14 days and age (r = −0.19, p = 0.02), and a weak positive correlation with mFS (r = 0.24, p = 0.005) and HHS (r = 0.25, p = 0.003). Median DMED in patients with thin SAH (mFS 1-2) and in those with thick SAH (mFS 3-4) were 101 mg and 209 mg; the distributions in the two groups differed significantly (Mann-Whitney U = 1460, n1 = 48, n2 = 87, p < 0.05 two-tailed) (Figure 2).

**Figure 3:**
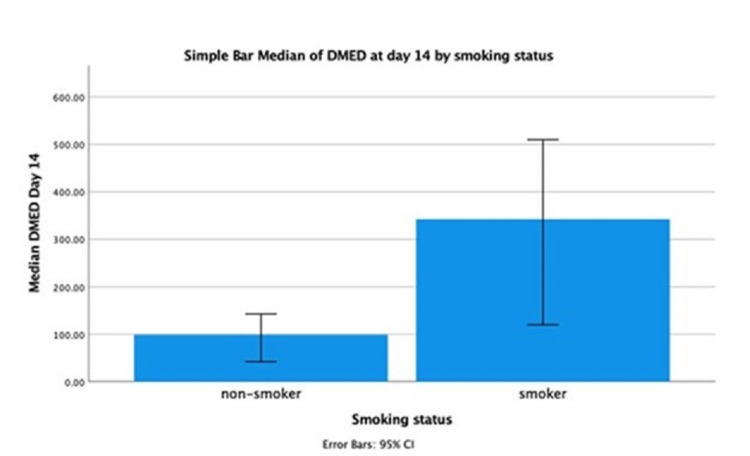
Smoking status and median DMED requirement. DMED: daily morphine equivalence dosage.

Steroid usage was associated with an increase in 14 days opiate utilization (r = 0.4, p = 0.0001). In addition, there was no statistically significant correlation between CSF WBC, RBC, protein, and lactate level, and opiate utilization. Over 14 days, smokers (median = 342.5 mg) significantly used more opiates compared to nonsmokers (median = 98.75 [round up to 0.1 mg], U = 290, p = 0.007) (Figure 3).

Patients with peri-mesencephalic SAH (median = 82.5, min = 0, max = 1264.75) required less morphine equivalence compared to aneurysmal SAH (median = 208.75, min = 0, max = 1167.75) (U = 1283, p = 0.001). There was a trend towards a higher DMED amongst patients who underwent aneurysm coiling (median = 320 mg) compared to aneurysm clipping (median = 186 mg); however, this was not significant (U = 538, p = 0.08). In multiple regression analysis, smokers, aneurysmal SAH, higher mFS, female gender, and younger age had significantly higher DMED usage [(3, 77) = 7.69, p < 0.05, R2 = 0.23] (Table 2).

**Table 2:** Multiple regression analysis. *** Significance at 95% level. HH: Hunt and Hess scale; DMED: daily morphine equivalence dosage; mFS: modified Fischer scale.

Constant	−5.32 (4.08)
Perimesencephalic/aneurysmal	6.01^***^ (1.26)
Age	0.10^***^ (0.05)
DMED over 14 days	0.01^***^ (0.002)
HHS	2.21*** (0.93)
mFS, gender, presence of hydrocephalus	1.93*** (0.62), 0.83 (1.20), 0.13 (1.25)
R^2^, number of observations	0.43, 123
Constant	354.404 (98.676)
Perimesencephalic/aneurysmal	125.867^***^ (47.422)
Age	−4.574^***^ (1.763)
Smoking status	146.545^***^ (60.243)
HHS	3.643 (45.232)
mFS	13.61 (24.30)
R^2^, number of observations	0.23, 80

**Figure 4:**
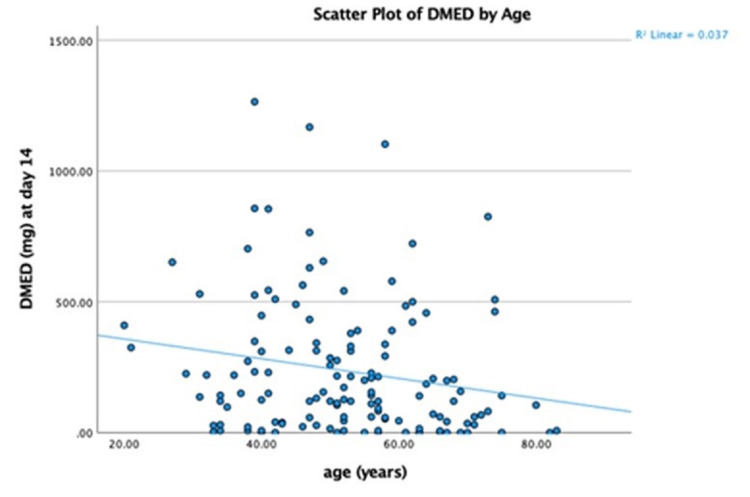
DMED correlation with age. DMED: daily morphine equivalent dosage.

**Figure 5:**
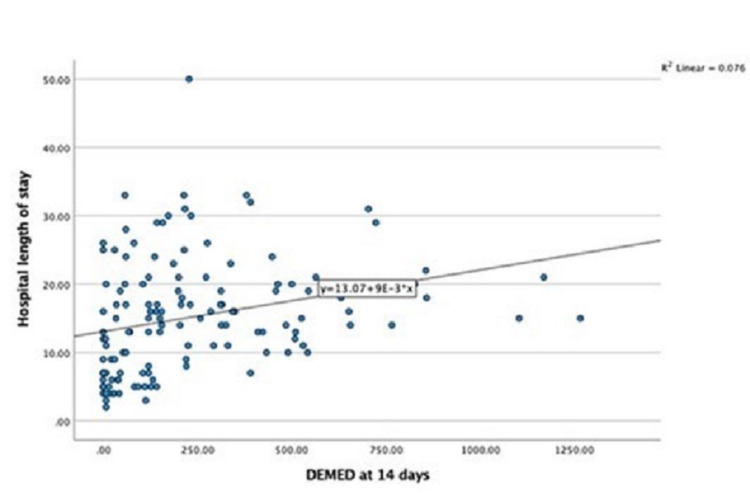
DMED correlation with hospital length of stay. DMED: daily morphine equivalent dosage.

There was a positive correlation between the amount of opiate used in the first 14 days of admission and the length of stay in the hospital (r = 0.28, p = 0.001; Table 1). Multiple regression was utilized to predict hospitalization length of stay from gender, age, HHS, mFS, presence of hydrocephalus, cause of SAH, and amount of opiate usage over 14 days. Older age, higher HHS and mFS, aneurysmal SAH, and higher DMED utilization significantly predicted hospital length of stay [F (7, 123) = 12.6, p < 0.05, R2 = 0.4] (Table 2 and Figures 4, 5).

A total of 124 patients (83.2%) had an outpatient follow-up, and 48 patients (42%) suffered from headaches during their outpatient follow-up within three months of discharge; however, only six patients (5%) were still on prescribed opiates (Figure 1).

There was a significant association between the amount of opiate used in the first 14 days of admission and the rate of post-discharge headache (288 mg vs. 136 mg, p = 0.002). There was no significant difference in the rate of post-discharge headache for males vs. females, distribution of age, presence of hydrocephalus, IVH, smoking status, or coiling versus clipping.

## Discussion

Opiate usage as analgesia in SAH is usually avoided due to its sedating side effect, which can mask the neurologic examination [9]. In addition, opiates can suppress respiratory drive, leading to hypercapnia, which could increase intracranial pressure [10]. However, despite its adverse effects, opiate consumption in this patient population remains very high. Similar to what has been previously described in the literature, we found that despite significant utilization of opiates, headache related to SAH remains poorly controlled [6]. Almost all of our patients required a significant amount of opiates, with a median DMED utilization of 142.5 mg (min = 0, max = 1264.75 mg). Moreover, about half were still suffering from headaches at the three-month follow-up. Previous studies have shown that patients with SAH suffer from severe headaches, which remain refractory to analgesics. A recent study noted that patients continued to suffer from SAH-related headaches for years following the event [7]. Moreover, patients who suffered from headaches due to SAH were more socially impaired, had more sleep disturbances, and increased stress and anxiety levels compared to patients with SAH who did not suffer from headaches [7]. Additionally, there was a significant difference in physical health composite scores amongst the two groups [7].

We evaluated the factors associated with opiate consumption. Given that inflammation induced from blood degradation products is thought to be one of the pathogenesis of headaches in SAH, we evaluated the association of CSF WBC, RBC, protein, and lactate with opiate usage. However, we did not find a statistically significant correlation between the CSF profile and opiate usage at 5% significance. However, elevated CSF protein was associated with post-discharge headache. Moreover, steroid usage was associated with an increase in 14-day opiate usage (r = 0.4, p = 0.0001). We would have expected that steroids would decrease inflammation, hence decreasing headache intensity and opiate usage. However, this could be the result of prescribing steroids for patients with more severe headaches, not responding to high doses of opiates. It remains unknown if steroids help with decreasing headache intensity, even though it is frequently used in clinical practice. We found that thick (defined as greater than or equal to 1 mm) SAH was associated with more median DMED usage. This was described as a correlation between Hijdra score and severity of headache [6]. In addition, perimesencephalic SAH required significantly less median DMED compared to aneurysmal SAH. This could be due to the different pathogenesis of injury. Perimesencephalic SAH is thought to be due to venous bleeding [11,12]. It is unknown why perimesencephalic SAH follows a more benign course compared to aneurysmal SAH, with less progression to delayed cerebral ischemia and better prognosis [12]. One possible explanation is that arterial and venous blood have different reactive oxygen species [13]. Oxidative stress is thought to be involved in the pathogenesis of cerebral vasoconstriction and headache in SAH [14].

In addition, similar to a previous study, we found a significant negative correlation between age and median DMED usage for headache management [3,6]. However, there was no association between gender, race, and the presence of hydrocephalus with opiate utilization for headache management during their hospital course.

Moreover, we evaluated the association between opiate usage in the first 14 days and length of hospital stay. We found that mFS, aneurysmal SAH, and amount of opiate usage significantly predicted the length of hospital stay. This could be due to the sedating adverse effects of opiates, causing alteration in the neurologic examination and leading to unnecessary testing and longer hospital length of stay.

A recent study revealed that a significant proportion of patients suffer from substantial headaches years after discharge [6,7]. Similarly, we found that 42% of patients followed within three months of discharge still suffered from headaches; however, only 6% were still using opiates for pain management. The small percentage of patients that were still on opiates within three months of follow-up could be due to strict opiate prescription policies since the emergence of the opioid pandemic. We also noted that there was an association between inpatient opiate usage and the incidence of outpatient headaches. It is likely that opiates cause transient pain relief; however, it does not target the pathogenesis of headache due to SAH; as a result, it does not have sustained effects. Other factors such as gender, age, presence of hydrocephalus, smoking status, or treatment modality were not predictors of headache incidence in the outpatient setting. This is similar to another study that showed no difference in the incidence of long-term headache among endovascular versus surgical treatment [7].

This study carries limitations worth mentioning beyond its retrospective design. Although we excluded patients that were intubated for more than 48 hours, we could not control the usage of opiates for reasons other than headache. In addition, the outpatient record lacked details about the headache intensity at three months follow-up.

## Conclusions

Even though patients admitted with SAH require a large amount of opiate for headache management, this did not lead to more opiate consumption in the outpatient setting. However, patients were still suffering from headaches at three months follow-up. In addition, high opiate consumption is associated with increased hospital length of stay. Studies are needed to identify opiate sparing analgesics that target the pathogenesis of headaches in this patient population.
